# Scenting Entertainment: Virtual Reality Storytelling, Theme Park Rides, Gambling, and Video-Gaming

**DOI:** 10.1177/20416695211034538

**Published:** 2021-08-23

**Authors:** Charles Spence

**Affiliations:** Crossmodal Research Laboratory, University of Oxford, Oxford, UK

**Keywords:** scent, entertainment, gaming, gambling, virtual reality, theme parks, multisensory storytelling

## Abstract

There has long been interest in both the tonic and phasic release of scent across a wide range of entertainment settings. While the presentation of semantically congruent scent has often been used in order to enhance people’s immersion in a particular context, other generally less successful attempts have involved the pulsed presentation of a range of scents tied to specific events/scenes. Scents have even been released in the context of the casino to encourage the guests to linger for longer (and spend more), at least according to the results of one controversial study. In this narrative review, I want to take a closer look at the use of scent in a range of both physical and digital environments, highlighting the successes (as in the case of scented theme park rides) and frequent failures (as, seemingly, in the context of scent-enabled video games). While digitally inducing meaningful olfactory sensations is likely to remain a pipe dream for the foreseeable future, the digital control of scent release/delivery provides some limited opportunities to enhance the multisensory experience of entertainment. That said, it remains uncertain whether the general public will necessarily perceive the benefit, and hence be willing to pay for the privilege.

## Introduction

There has been a long history of people trying to introduce a scented element into cinema entertainment (e.g., see Gilbert, 2008; Spence, 2020b, for reviews) as well as a range of other live-performance settings, including the theatre, opera, and so on (see Spence, 2021a, for a recent review). For decades now, ambient scents have, on occasion, also been incorporated into museum exhibits and art galleries too (e.g., for a review, see Spence, 2020a), as well as in other entertainment venues, such as theme parks including the Epcot Centre in Florida (Lukas, 2008) and Disneyworld in California (Mack, 2014). However, it has been the various attempts to digitally control the release of scent in the context of the cinema that perhaps comes closest to use of scent in video games or other forms of digital home-entertainment, such as watching the TV, that are the focus of this narrative review.^1^ I would like to summarize the sometimes successful, though more-frequently unsuccessful, introduction of scent into a range of entertainment settings including both physical, though more often digital, spaces/environments. In particular, this review will focus on the costs and benefits of introducing scent in a variety of virtual reality (VR) storytelling applications (e.g., Delaunay, 2014; Park et al., 2002; Ranasinghe et al., 2018), in theme parks (Mack, 2014), in the casino (i.e., gambling; Hirsch, 1995; see also Lynch et al., 2020), and in video-gaming (e.g., Ranasinghe et al., 2019).^2^

At the same time, however, I would also like to bring out some of the peculiarities of the home-entertainment/gaming setting that make it a qualitatively different proposition from these other, much more public, forms of entertainment as far as the introduction of scent is concerned. For one thing, it is simply much harder to know how the action will unfold in the context of gaming, say, thus making it much harder to optimize the sequencing and timing of scents that are tied to specific events, scenes, or places. This, it is worth stressing, turned out to be a key factor in the limited success of scent’s evanescent use in cinema (see Spence, 2020b, for a review).

## Scent-Enabled Virtual Reality

**Figure 1.**
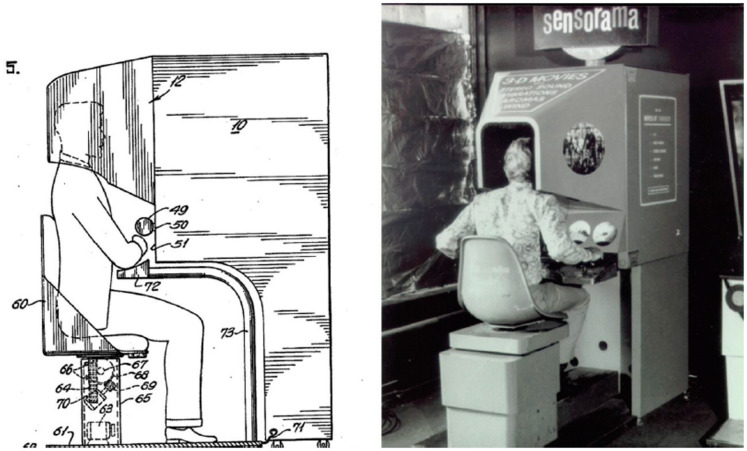
Preparatory sketch (on the left) and photograph (on the right) of the Sensorama Simulator patented by M. L. Heilig (1962). This invention is widely credited as being the first simulator designed to stimulate multiple senses. Figure reproduced from Heilig (1962, Figure 5), Patent 3,050,870.

Researchers have long been interested in the incorporation of scent into a range of VR applications (see Barfield & Danas, 1995; Cater, 1992, 1994; Heilig, 1962), starting with North American cinematographer, Morton L. Heilig’s (1962) *Sensorama* simulator (see Figure 1). Although never a commercial success, this early attempt to introduce scent into a multisensory entertainment setting was considered highly innovative at the time.^3^ As described by Heilig in his original patent application from January 1961: “The present invention, generally, relates to simulator apparatus, and more particularly, to apparatus to stimulate the senses of an individual to simulate an actual experience realistically.” The device consisted of a machine in which the user was presented with 3D images, various scents, stereo sounds, wind effects, and vibrations. One of the very few films ever made especially for Sensorama simulated a motorcycle ride through Brooklyn. The sense of presence was enhanced in this case by introducing various other sensory cues including the wind that blew through the user’s hair, the appropriate sounds and, most importantly for present purposes, the smells of the city. The bumps in the road, associated with driving over cobblestones, were simulated by means of the vibrating seat on which the user perched. The aromas of jasmine and hibiscus were apparently released as the driver passed a flower garden, and the scent of baking pizza as one passed an Italian restaurant in Brooklyn (Rheingold, 1991, Chapter 2). Unfortunately, however, this early attempt to bring scent to VR never received sufficient funding, nor commercial interest, to warrant scaling-up to ever represent a viable commercial proposition (Kaye, 2004).

A rather less optimistic view about scent’s role in the future of Augmented Reality (AR) appeared in The Ultimate Display, Ivan Sutherland’s highly influential article on the future of computing, first published in 1965. Sutherland certainly did not see anything on the horizon as far as the digital delivery of scent or taste was concerned, writing more than half a century ago now that: “So far as I know, no one seriously proposes computer displays of smell, or taste” (see Sterling, 2009; cf. Craig et al., 2009). In the contemporary era, it has been suggested that VR may have a role in both entertainment and training applications. Interestingly, even in his original patent application, Heilig was already highlighting the potential benefits for a variety of training applications of engaging more senses. There has been much interest in the use of scent to enhance the sense of presence/immersion (Jones et al., 2004; Jung et al., 2020; Munyan et al., 2016; Zybura & Eskeland, 1999; though see also Slater, 2004), and consequently the beneficial effects of various military/battlefield surgical training VR applications (e.g., Herz, 2007; Vlahos, 2006). An olfactory element has also been introduced into the training that firefighters and those professionals hoping to work in a number of other challenging environments receive (e.g., Kaye, 2004; Washburn et al., 2003). Others, meanwhile, have considered the possible benefits of introducing olfactory cues into everything from helping to transfer useful information in the case of tele-surgery (Keller et al., 1995) through to enhancing data visualization (Washburn & Jones, 2004). Here, though, it is important to highlight the fact that humans are visually dominant (see Hutmacher, 2019, for a review). As such, one might wonder how much olfaction is really likely to add to the multisensory experience of VR, given that it is such a visual media to begin with (see also Gallace et al., 2012; Heilig, 1955/1992).

In her 2007 book, *The scent of desire: Discovering our enigmatic sense of smell*, Rachel Herz notes that the American military have long been pumping money into VR simulations, incorporating what is known as a Scent Collar, patented in 2009 by Jacki Morie, a researcher working at the USC Centre for Creative Technologies at the University of Southern California (Anonymous, 2009). At the time Herz was writing, a 10-channel version of this scent delivery system was apparently in development. It has been suggested that the addition of a scent collar to standard VR equipment (e.g., goggles offering a stereoscopic view, headphones providing binaural sounds, and movement sensors) might help to create a more immersive multisensory environment in which soldiers/surgeons can be prepared for the kinds of situations that they may subsequently be expected to encounter in a war zone (or humanitarian emergency): Be it the sweet smell of a decaying corpse, or the smell of a cigarette that gives away the presence/position of an enemy combatant (the text here adapted from Vlahos, 2006).

There has also been interest in the use of scent (sometimes combined with haptic cues) in various therapeutic and rehabilitative applications of VR, such as to help those recovering from Post-Traumatic Stress Disorder (PTSD; e.g., Aiken & Berry, 2015; see also Covarrubias et al., 2015; Chen, 2006; Rizzo et al., 2006; Serrano et al., 2016). Several research groups have been investigating the incorporation of scent into a range of pre-travel/tourism applications (what is sometimes referred to as “virtual tourism”; e.g., Flavián et al., 2021; Hopf et al., 2020; Martins et al., 2017). For instance, according to Flavián et al., semantic congruency can play an important role in determining the efficacy of the scents in such applications. These researchers found that congruent/pleasant ambient scent influenced people’s affective and behavioural reactions. In their VR simulation, these researchers used the smell of coffee as the congruent pleasant smell for Venice, while grass was used to match the Cliffs of Moher (and was rated as equally pleasant). Importantly, however, the results that have been published to date have been rather mixed. That is, no particular benefit (in terms of enhancing presence) of adding scent was reported in Hopf et al.’s study. By contrast, the addition of both olfactory (the scents in this 6-minute experience were described as the typical smell of the ocean and the rainforest) and tactile inputs (wind, rain, etc.) to the destination-based VR experience resulted in a significant increase in the user’s intention to recommend the destination to others (Hopf et al., 2020). This result contrasting with Flavián et al.’s recent laboratory-based study which, as we have just seen, evidenced a significant benefit of adding a semantically congruent pleasant scent (see Ghinea & Ademoye, 2012, on the role of congruent/incongruent scents). And, as might have been expected, there is also interest from the porn industry in harnessing the VR scent technology (Cole, 2017).

More relevant to the themes of the present review is the Kyongju VR theatre, a large venue built for the Kyongju World Culture EXPO 2000 in Korea that could accommodate an audience of up to 651 people. According to Park et al. (2002), five different fragrances were delivered by means of air ducts under the floors all controlled by computer for this example of large-scale multisensory storytelling. There was a 15-minute experience called “Journey into the breadth of Seorabol.” In terms of the mechanism of scent release, the fragrances were dropped onto a heated plate and released into the air ducts and the ventilation system, thus carrying aroma throughout the entire theatre. The pine scent released while the mountain scene was displayed was apparently the most effective.^4^ The identity of the other four scents are not mentioned. However, it is worth noting that in large-scale (VR) situations, it can be hard to capture/control the realistic time course of the diffusion of olfactory stimuli throughout a space (see Spence, 2020b, on this theme). At the end of the experience, the audience could press on the left or right buttons on a keypad to control the movement of an immersive screen full of butterflies. Furthermore, given the large-scale public (rather than private) setting of the Kyongju VR theatre (which ran for more than 2,500 shows), one might almost be tempted to consider this as an example of scented cinema. That said, it is worth noting how previously it has been suggested that the computerized aroma balls developed by NTT in Japan, and trialled in the setting of the cinema, might actually have worked better in the home environment, where they could either be sold or rented alongside the matching DVDs (see Fujiwara, 2006). All this to say that there may sometimes be a fruitful crossover between the introduction of scent into the cinema/VR theatre setting and home-entertainment situations.

Described as “a VR game” by Kerruish (2019, p. 32), Season Traveller was rather an innovative example of multisensory storytelling/entertainment, incorporating a mechanism, or sensory-enabling technology (SET; Petit et al., 2019), that could deliver wind, temperature, and smell cues on a head-mounted Samsung VR headset (Ranasinghe et al., 2018). On donning the headset, the user was taken on a 120-second “mystical balloon ride” through four seasonal landscapes, each lasting for 30 seconds. A four-chambered air-pump powered scent-delivery mechanism delivered a different scent to match each season: Jasmine for spring, lemon for summer,^5^ cinnamon for autumn, and a cooling mint scent to match winter. The results suggested that the addition of a sense enhanced the user’s sense of presence in this VR application while combining multiple senses appeared to enhance it still further.

According to Chen (2006): “scents are extremely evocative in the virtual world, they can shift attention, add novelty, enhance mental state and add presence” (p. 580; see also Covaci et al., 2018; Gallace et al., 2012). That said, one of the key findings from the laboratory-based scent-enabled VR research that has been published to date has been that while unpleasant scents tend to increase immersion, positive and neutral scents often show little effect (e.g., Baus & Bouchard, 2017; Ghinea & Ademoye, 2012; Ischer et al., 2014; though see also Toet et al., 2013). Indeed, according to the title of a 2019 paper by Baus et al.: “Exposure to a pleasant odour may increase the sense of reality, but not the sense of presence or realism.” According to the latest research, scents, no matter whether pleasant or unpleasant may enhance the memorability of virtual visual environments (see Sabiniewicz et al., 2021, on pleasant scents and Tortell et al., 2007, on the memory-enhancing effects of the presumably unpleasant synthetic smell of a murky, smoky, swampy culvert). Such observations have been linked to the special status of unpleasant smells, including the fact that we never seem to adapt to them. The suggestion, in this case, being that this is because they may potentially be dangerous (Spence, 2002). The everyday example of this being that we all adapt (and so become functionally anosmic) to the smell of our own home, until we come back from a long holiday (Dalton & Wysocki, 1996), whereas those living next door to the animal farm never do get used to the smell (e.g., Anonymous, 2018; Matheny & Honoré, 2011; Spence, 2021b). Figuring out if/how scent can be integrated into VR is likely to be financially relevant given that according to Virtual Reality Market (2016) the field is estimated to grow from $1.37 Billion (USD) in 2015 to $33.90 Billion (USD) by 2022.

As well as considering the importance of odour pleasantness, it is also worth noting that intensity is a relevant dimension of olfactory experience, sometimes being related to pleasantness and/or emotional impact of scent (Bao & Yamanaka, 2016), but also, on occasion, to phenomenal quality, with a small number of odourants being associated with a different odour source at different stimulus intensities (Koza et al., 2005; Kruger et al., 1955; Mesfin et al., 2020). Perceived odour intensity is obviously also influenced by distance from the source of scent delivery as well as the degree to which the scent-enabled device targets the direction of scent (see Aroma Shooter, 2020). As we will see later, there are situations in which the efficacy of the scent appears to be tied to its intensity (see Hirsch, 1995).

## Incorporating Scents Into Theme Parks

According to a book chapter by Adam Mack (2014), the multisensory atmosphere of theme parks such as Disneyland in California is very carefully controlled and orchestrated, and that includes the targeted release of specific scents. Meanwhile, at Disneyland, sweet candy scents are diffused throughout the site (e.g., on Main Street, the main thoroughfare that all visitors pass through on entering the park).^6^ However, in this case, the scent may well be used as much to boost food and beverage sales as to help enhance the visitor’s sense of immersion in the experience (see Spence, 2015a, for a review of the use of food scents in marketing food; see also Gilbert & Firestein, 2002, on the importance of olfaction to marketing/branding). However, beyond the ambient scenting of the public spaces/thoroughfares, scent is also deliberately incorporated into a number of the themed rides as well.

According to Fjellman (1992, pp. 72, 333, 362), Walt Disney World in Orlando, Florida has been using its patented Smellitzer aroma cannons for decades (see McCarthy, 1986). Meanwhile, at Disneyland’s California Adventure theme park, a gentle smell of citrus has for many years now been spritzed over visitors during a ride at the point at which they appear to be soaring over a grove of orange trees (Legro, 2013). Notice how, in this case, the scent is semantically congruent with the scenery. Indeed, this pleonastic use of scent would always appear to be the case in the setting of the theme park. Thus, just as in the majority of museum displays (Spence, 2020a), the visitors get to smell exactly what they see (or, more likely, a synthetic reproduction of it; Eco, 1985).

According to one Disney blogpost:

The smellitzer operates like an air cannon, aiming the scent up to 200 feet across a room toward an exhaust system. Guests traveling on the moving vehicles will pass through the scene as the appropriate scent drifts across their path. Regulated by computer, the scent can be triggered for a fresh aroma just prior to each vehicle's arrival. (Anonymous, 2014)^7^

Interestingly, a couple of years ago, a patent was registered for a modified version of the Smellitzer allowing for the gradual introduction of a scent rather than delivering it all at once, as well as allowing one to deliver multiple scents (Reichow et al., 2019; see also Jaalin, 2019). However, as yet, it is not clear what use these new scent-delivery systems will be put to. That said, the opening paragraph of the patent application stresses one possible use of scent to be congruent with the visual entertainment, and later the suggestion is made that multiple scents could be presented sequentially to be congruent with sequential elements in a story.

In a 2019 blogpost, McCormack listed 17 different scents that Disney fans might recognize. They included the smell of the orange groves on Soarin’ Over California, the African grasses on Soarin’ Around the World (at Walt Disney World’s, Epcot Centre in Florida), and the cannons and salty water at Pirates of the Caribbean (at Walt Disney World’s, Magic Kingdom). Meanwhile, according to Anonymous (2014):

Some of the most unusual scents will be in the Land pavilion at Epcot Center. Here, the visitors will experience tropical vegetation, rain forests, deserts; some of the great terrain found on Earth. Of course, Disney “Imagineers” plan to supply all the appropriate smells. Guests traveling through a farming scene may detect a faint animal smell. In another scene, an orange grove will smell like the real thing. Still another effect calls for the smell of damp earth.

There are a couple of salient points to note here: First, the fact that the passengers on the rides move continuously (and entirely predictably) through the different spaces in the pavilions helps to resolve the longstanding problem of the build-up of different scents that often stymied previous attempts to add digitally controlled scent sequences to films shown at the cinema (see Spence, 2020b). Second, it is also striking how all of the examples described earlier, the scents are straightforwardly semantically related to (i.e., congruent with) the scene, or objects therein. That is, the aim would appear to be all about matching the surroundings to create a more believable, and presumably immersive, experience (Anonymous, 2014). There is, for example, no attempt to tell jokes by means of scent as was highlighted as one of the more interesting features of the (least unsuccessful) examples of scented cinema (see Spence, 2020b, for a review, and see Banes, 2001, for the multiple different uses of scent identified in the context of live performance). However, I have been unable to find any research that has directly compared people’s enjoyment of scented versus unscented rides.

This is also true of the 4D Pacific Visions Experience at the Aquarium of the Pacific in Long Beach, California delivered by SensoryCo Scenting System. According to the company’s website:

“ocean” and “fresh cut grass” cartridges are triggered to release fragrance when programmed to coordinate with the various scenes in the film presentation. When the sights and sounds are combined with the 4D effects of wind, motion and scent, human senses are stimulated for visitors to see, feel, and smell … leaving a lasting impression. (Anonymous, n.d.)

Targeting claims around the enhanced memorability of the experience would appear to be a good idea given the evidence that has been published to date in the literature (Covaci et al., 2018; Dinh et al., 1999).

## Scented Slot Machines

One of the most successful uses of scent in the context of gaming, or rather gambling, was reported by Hirsch (1995; see also Miller, 1993). Hirsch reported that gamblers spent 45% more (*p* < .0001) on slot machines when a particular artificial scent was used in one section (comprising 18 quarter slot machines) of the Las Vegas Hilton casino as compared to non-scented sections in the same area, or else to another section where a different scent had been used. It also led to a significant increase relative to takings on the preceding and following weekends. The scent was present for 48 hours starting midnight Friday. Unfortunately, the identity of this most efficacious scent was never disclosed.^8^ However, given that the gamblers were not quizzed about how much they were enjoying themselves, nor how immersed they were in their gaming, it is unclear whether or not their gambling enjoyment was also enhanced by the scent. Those who support the use of ambient scent in retail environments often suggest that a well-chosen scent primarily enhances the consumer’s experience which, in turn, may lead them to linger for longer, and thus spend more (i.e., rather than scent having a direct effect on spending; see Spence et al., 2014). Much the same point can presumably be made in relation to Hirsch’s study. It is perhaps also worth noting that this intervention did not require any particular high-tech solution to deliver the scent. Instead, Hirsch used dispensing devices (though no more detail provided), with the author noting that takings were higher on Saturday when the scent was stronger.

Should Hirsch’s (1995) findings be reproducible (see Wilkie, 1995), no mean feat given that we do not know which scent was used, one might only wonder at the possibilities around the scenting of online gambling, given what a huge business that has become in many countries in recent years. To give the reader some idea, the global online gambling business was estimated to be worth $40 billion in 2020 and is expected to generate revenues of almost $75 billion by 2023 (Anonymous, 2020). What is more, as casino gambling increasingly moves onto smartphone devices (and hence into the home environment), the financial benefits of sending out a plug-in scent dispersal device (such as the Scentee, https://scentee.com) loaded with Hirsch’s proprietary potion to even a tiny proportion of the estimated 1.6 billion online gamblers worldwide, would likely be an investment that was well worth the cost of postage and packaging to the already phenomenally successful online gaming enterprises. This assuming that there is no law against such olfactory manipulation which, as far as I know, there is not!

Beyond simply ambiently (and tonically) scenting the entire gaming floor, other commentators have wondered about the possibility that slot machines could one day be upgraded to release the musty smell of money and perhaps also ripe cherries when someone hits the jackpot (Spence, 2002). Such pulsed release of scent would likely capture attention, and rapidly become a very positively valenced smell, much like the often-discussed new car smell (Aikman, 1951; Spence, 2021b). Alternatively, however, one might also consider following the lead of the North American bank that considered scenting the money dispensed from its ATM cash machines with mint, in the belief that this would make it smell fresher than that of their competitors (Miller, 1993). On the downside, however, notice how these latter suggestions would all presumably require each and every slot machine to be wired for scent.

## Scented Television

Researchers have also been interested in the addition of a fragrant element to our small screen entertainment, be it the TV (Burgess, 2016; Poniewozik, 2000; Tan, 2012), or nowadays, smaller still the smartphone screen (Nakamoto & Yoshikawa, 2006; Sebag-Montefiore, 2015), again perhaps via plug-in devices such as the Scentee (Braun et al., 2016; Collins, 2014; see also Braun, 2019). In 1999, the Cartoon Network launched Smelly Telly (May 8, 1999; http://cartoonnetwork.com/promotion/smellytelly/), with Smelly Telly Rub and Sniff Card. Numbers appeared on the TV screen on select episodes of Cow and Chicken episodes. The viewers at home were supposed to rub the matching spot on their cards. Meanwhile, in the same year, four episodes of The Wild Thornberry on Nickelodeon cable TV were also accompanied by scratch ‘n’ sniff (Paterson, 2006). That said, although scratch and sniff was a commercial failure when introduced at the cinema (by John Waters, in 1981, when he introduced the world to Odorama with his movie Polyester), those who have tried to revive this approach (and used the Odorama name) have been slapped with cease and desist orders by Waters’ lawyers at New Line Cinema (see Gilbert, 2008, p. 166).

If Hamid Arastoopour, Firooz Rasouli, and Ali Oskouie of the Illinois Institute of Technology (IIT) had had their way, Web sites would long ago have been tickling the nostrils of those who were online. The three scientists patented the design for a Tele-Aroma Drive that has been proposed to deliver the first web-smells (TAD; Brooks, 2000; Jacobson, 1999). As Jacobson noted/predicted:

Eventually, as computers and televisions converge into a single piece of equipment, TAD technology might make possible a modern incarnation of “Smell-O-Vision,” a process tried with little success in movie theaters in the 1950s. Under the new system, viewers could watch a movie on their home TV and smell the same pine forest or the same cloud of gunpowder as the character on screen.

In 1999, at around the same time, a reporter from Wired magazine described how they got to experience Digiscents *i*Smell system (Platt, 1999). In particular, the reporter experienced scented movie clips from *The Wizard of Oz* (notably one of the very first successful colour movies, released in 1940; Spence, 2020b), with the aroma of cedar as Dorothy and her companions entered the forest, and the scent of wood smoke as the witch stirred her potion over the fire. Digiscents Inc. was founded by Joel Lloyd Bellenson and Dexter Smith in 1999, and had esteemed sensory scientist such as Avery Gilbert (2008) on the board of advisors (deLahunta, 2003). Unfortunately, however, the company folded in Spring 2001 when the requisite venture capital funding was not forthcoming, and no hardware vendors materialized (despite seducing many commentators and companies along the way; Bosner, 2001). With the benefit of hindsight, Time magazine rated Digiscents iScent as one of the 50 greatest failures of all time (Fletcher, 2010).

Elsewhere, it has been suggested that the smell of fresh cut grass might help enhance the football fan’s experience of watching a game (see Spence, 2002; cf. Nakamoto & Yoshikawa, 2006; Ramic-Brkic et al., 2009). In this case, though, one might again question the need for the digital control of scent delivery. That is, a straight-forward grass-scented air freshener would presumably do the trick, given that they playing surface does not change much during the course of the 90-minute game.^9^

“If you watch leather-lunged TV megachef Emeril Lagasse, you've probably heard him lament the limitations of his medium: ‘Oooh! I can't wait till we get Smell-o-Vision so you can smell this at home!’” (Poniewozik, 2000).^10^ More recently, researchers working at the University of Sussex have suggested to enhance the sensory range of TV. According to one press report: “ … this is ‘9D’ television … But why 9D? The setup combines vision, touch, sound, smell and all five tastes (sweet, sour salty, bitterness and umami), the team explains.” … although later in the article we find another member of the group, Dmitrijs Dmitrenko, admitting that: “Taste hasn’t been developed yet, but could be made possible by making viewers drink small amounts of flavoured liquid at certain times during a film.” So placing this firmly in the mixed reality (MR) category.^11^ Furthermore, according to the journalist, the olfactory element was described as “indistinguishable” (Burgess, 2016), suggesting that more work is needed to develop something that might attract the punters.

Interestingly, however, the enthusiastic press response, once again highlighted the excitement that surrounds attempts to extend the bandwidth of TV, not to mention other forms of entertainment. Thinking back, though, it is perhaps worth bearing in mind how sound, when it was first introduced to moving pictures was by no means universally popular. While the much more enthusiastic suggestion that touch could be added to cinema (as memorably captured by Aldous Huxley in his often quoted line from *Brave New World* about going to the “Feelies”; Huxley, 1932)^12^ never did succeed (see Frost, 2006). So why, then, should we think that the case with scent would be any different? While many in the HCI community would appear to have focused their energies on the question of how to digitally deliver tactile stimuli, and digitally control the release of olfaction, there has been far less thought given over to the question of why. To your present author, the question of what exactly the USP (unique selling point) of scent-enabled home entertainment is, or might be, has not yet been made clear.^13^ One sometimes wonders if it might simply to try and address the problem that it can be hard to impress the youth, as captured by the following quote:

Most designers have gotten to the point in production where the decision is made to hit the viewer with everything they’ve got. The big sounds, the dramatic slam of music from the dead silence, the sudden appearance of the beast. And the kids sit there saying ‘been there … done that … ho hum … (Ralph Thomas, quoted in Anonymous, 2002)

## Scenting Video Games

Scent has been introduced into a variety of digital gaming contexts, including both traditional video games and, more recently, VR gaming. Writing a little over two decades ago in The Guardian newspaper, Brooks (2000) noted that:

Rob Dyer, president of Eidos Interactive, says the demo pack has convinced him that smell has huge possibilities; Eidos programmers are already playing with the smell of Lara Croft. Eventually, you'll smell those dank caves as you roam through the darkness. Race games will get a new dimension with the smell of burning rubber to spice up your living room.

Another suggestion was that players might one day use bad smells in order to disrupt their opponent’s gameplay: “To the gaming world, of course, that's just another opportunity. Multi-player games that can ruin your opponent's concentration with a well-timed stink bomb? Now that's progress” (Brooks, 2000). However, it is worth stressing that the widespread introduction of scent-enabled games has been predicted to be “just around the corner” for more than two decades now (Anonymous, 2008; Brooks, 2000); It still hasn’t been realized.^14^

At the same time, however, one might want to question why it should be considered necessary, or even desirable, to incorporate a scented element into a digital video gaming context in the first place, given that no one seemingly ever thought it either desirable, or necessary, when physical game (e.g., board games) were all there was.^15^ Would a game of Monopoly be enhanced simply by giving one’s properties a signature scent, or pulsing out the scent of money whenever a player passes go and collects £200 (in the British version of the board game)? Or would a game of Operation be more exciting if Cavity Sam were to release an antiseptic/hospital smell during play (perhaps something like TCP, a very strong smelling traditional antiseptic lotion)?^16^ The same can be said about the early arcade video games, such as Space Invaders, or Asteroids—surely no one would really think that releasing a synthetic “gunpowder, hot metal, welding” smell, to match the reports that have come back from astronauts about the smell of space (New York Hall of Science, 2016) would be a good idea. The historic lack of an olfactory element in traditional board games, or traditional arcade video games, contrasts with the case of storytelling where multisensory approaches incorporating olfaction have sometimes been explored (Delaunay, 2014; Legro, 2013; Spence, 2020d).

Ranasinghe et al. (2019) developed a first-person 3D adventure video game that incorporated an optional LED and olfactory elements. These researchers conducted a small study in which 19 people played the game and rated their immersion/connection with it, either when simply playing the game as normal, or else when the LED and/or olfactory cues (namely, the scents of jasmine, pineapple, mango, and banana) were activated. The results of this small-scale proof of principle study hinted at the potential benefits of an olfactory element either when presented alone, or else when combined with the LED which illuminated the scent bottles. The results suggested that combining the extra sensory elements enhanced the player’s self-reported sense of presence (or immersion). However, as is so often the case in this kind of research, the sample size was small (and, when combined with the fact that no power calculation was reported to determine whether the sample size was sufficient to obtain meaningful results, it is a little unclear what can legitimately be claimed). What is more, around a quarter of the participants in this study reported that the fruity scents were not so distinct.

Another relevant example here is represented by Nosulus Rift, a somewhat bizarre odour-VR product created for Ubisoft's latest South Park game by Paris agency Buzzman and its product arm, Productman (Natividad, 2016). In the game, South Park: The Fractured But Whole … players are blessed with a unique superpower—magical farts. However, it is worth noting that only one scent was released from the face mask. Nosulus Rift was trialled at gaming shows (cf. Quick, 2011), and demonstrated to the gaming press, though never intended to be produced commercially. Indeed, it is noticeable how while a number of scented VR games have been demo-ed by various research groups at the HCI conferences such as SIGGRAPH, CHI, and so on (e.g., Mochizuki et al., 2004), as yet, there is little evidence that any of these proposed solutions have gained any traction outside of this particular environment. Before closing this section, it is interesting to consider how scent might play into more innovative gaming experiences such as, for example, offered by the much-hyped The Void. The latter is a gaming experience in which players don a pair of Oculus Rift glasses and then move through a series of spaces (Brennan, 2015; Jenkins, 2019).

However, while VR gaming has been touted as the next big thing for years now, for whatever reason it just has not ever really caught on (Adams, 2016; Jenkins, 2019). The latest failure in the Augmented Reality headset space was Magic Leap Inc. The company acknowledged defeat in the home entertainment market in 2020, after spending over US$3.5 billion of investors’ money in nine years (see Brustein & King, 2020). One intriguing smell-enabled attachment to commercial headsets does, however, look intriguing (FEELREAL, 2021), at least in terms of working across platforms, and being adapted for use with headsets.

Finally here, even should the technology be affordable (Magic Leap headsets cost around US$2,300 a set; Jenkins, 2019; while Microsoft Hololens Augmented Reality headsets are even more expensive; Bass, 2015) and functional, there would nevertheless still be physiological concerns related to negative health consequences, for vision and balance of extended use of such unnatural stimulation devices (as VR and AR), especially among children given that their perceptual systems are still developing (McKie, 2017). The discomfort and narrow field of view, as well as limits in social interaction that wearing such headsets may elicit also work against the technology. A quick look at the apparatus needed to deliver the Season Traveller by Ranasinghe et al. (2018) again makes it seem that the amount of kit that needs to be mounted on a user’s head may simply be too much for the benefits delivered.

## Unique Challenges and Opportunities Associated With Scent-Enabled Gaming

A wide array of different personalized scent delivery devices have been developed over the last quarter of a century or so (see Murray et al., 2016, 2017; Obrist et al., 2014; Obrist et al., 2016; Yanagida, 2012, for reviews). These include iSmell from Digiscents (Platt, 1999); the Aromajet, from Aroma Join; Aroma Shooter (Aroma Shooter, 2020); OSpace (Dmitrenko et al., 2017); ScentKiosk Scent Dispenser; Scent Scape (Anonymous, 2011); the Scent collar (Gallace et al., 2012; Herz, 2007); the Scentee (Braun et al., 2016), SpotScent (Nakaizumi et al., 2006), and so forth. Beyond these examples, there have also been a very large number of other technical solutions to the digitally controlled delivery of scent that have been proposed (e.g., Aravinda & Krishnaiah, 2013; Davide et al., 2001; Digital Trends, 2014; Herrera & McMahan, 2014; Hodson, 2013; Matsukura et al., 2013; McGookin & Escobar, 2016; Micaroni et al., 2019; Nakamoto et al., 2008; Ohtsu et al., 2009; Papin et al., 2003; Olorama, 2020; Quick, 2011; Ramic-Brkic & Chalmers, 2010; Seah et al., 2014; Suzuki et al., 2014; Watkins, 2002; Yanagida et al., 2004). All these references are cited in order to make the point that the technical challenges of controlling the digital delivery and spatial dispersion of scent have multiple solutions.

However, while the technological solutions available to digitize the delivery of the chemical senses have certainly come a long way over the last quarter century or so, it is fair to say that ambient fragrance still is not a widespread element in our everyday digital multimedia experiences (Kaye, 2004). Furthermore, with no standardization across the field, there is a danger that the consumer would need to buy a different smell interface for each new game/multisensory story.^17^ Another solution that looks intriguing is the Feelreal sensory mask (FEELREAL, 2021; see also Cakebread, 2017, for a Japanese version, Vaqso), though according to the website, Feelreal seemingly gives equal weight to its use as an aid to relaxation as to gaming and multisensory storytelling. Indeed, in the long-run, digitally controlled olfaction may turn out to have a more successful future in the context of sensehacking well-being (Spence, 2020c, 2021b) than necessarily in enhancing either gaming or storytelling.

One of the fundamental problems for all digital scent delivery systems is that no one has yet been able to figure out a way of reducing odour perception to some number of odour primitives. This problem was highlighted a few years ago in the following quote:

Turin believes that Smell-O-Vision has never taken off because, unlike colour TV, smell has no primaries that can be mixed to make endless combinations. “You cannot create an enormous palate of smells the way you can with [just three primary] colours,” he explains. “And that is a fundamental technological problem” (quoted in Sebag-Montefiore, 2015).^18^ This difficulty is one that early proponents of scented cinema, such as Heilig (1955/1992), simply failed to recognize.

The solitary nature of so much gaming contrasts with the shared public experience of museum’s/galleries, cinema/theatre, and so on (Spence, 2020a, 2020b, 2021a). This physical isolation (note that multi-player games are typically conducted remotely), together with the reduced distance between the player and their digital technology means that the targeted delivery of scent stimuli ought to be a little easier to achieve than when one is considering the introduction, and subsequent removal, of a sequence of scents from within large enclosed public venues such as movie theatres or opera houses (Spence, 2020a, 2020b, 2021a). At the same time, however, one of the other challenges in a gaming context is that it can be hard to know when exactly a scent should be released, whereas in the setting of the cinema, theatre, or theme park ride it can be precisely controlled thus allowing time for one scent to clear before next arrives, By contrast, a gamer’s progression is much less certain, and there might be a very real danger of olfactory overload or adaptation were a gamer to get stuck at a certain point in the game where there was a scented element to proceedings (cf. Kadowaki et al., 2007). And while Scratch n’ Sniff cards have occasionally provided an interesting opportunity to deliver personal scent in the context of the cinema (see Spence, 2020b, for a review), opera (Hone, 2006), and home TV setting (as we saw earlier), one challenge with doing the same in the case of gaming is that the players tend to be busy controlling joysticks and hitting the buttons, meaning that they may not have a hand free to scratch anything without interrupting their game-play.

Finally here, it should be noted that the problem of how to clear the air between successive scent deliveries (and so avoid a build-up of competing odours), which was one of the main reasons why scented cinema never took off (see Spence, 2020b, for a review), is likely to be of less concern in the case of personalized gaming. This is because in the latter case the delivery of scent can be targeted to the individual (i.e., rather than having to stimulate the entire audience, as in the case of scented cinema). Furthermore, for those systems where the scent is dispensed from close to the user’s nose, the amount of volatile chemicals that need to be dispensed in order to deliver an olfactory experience of a given intensity is also lower. Synchronizing scent delivery to the inhalation stage of the breathing cycle would further help reduce the total scent delivery needed (Kadowaki et al., 2008), though there is little evidence of the commercial applications of scent-enabled technology for the home having adopted this approach, as yet.

## Mixed Reality Solutions

Mixed reality solutions have long been suggested as one potential means of increasing immersion while at the same time dealing with the limited ability to deliver scent digitally (e.g., Dinh et al., 1999; Gallace et al., 2012; Hoffman et al., 1998). Mixed reality storytelling has delivered some intriguing, if labour intensive, experiences (e.g., see Pells, 2015). The problem being that they are not obviously scaleable. Researchers have recently started to investigate the potential to deliver digital taste in a gaming context too (Obrist et al., 2018; Vi & Obrist, 2018; see also Planet Licker: http://a-o.in/games/pl/; Murer et al., 2013), based on the idea of taste cues acting as primary reinforcers (i.e., sweet taste innately pleasant and bitter taste innately aversive). Despite the early state of gustatory-enabled gaming (Obrist, Gatti, et al., 2017; Obrist, Ranasinghe, et al., 2017), concerns about possibly encouraging obesity through rewarding players with sweet treats/rewards have already been raised. What is more, it is worth noting that digital taste, while possible, has so far proved to be a pretty “thin” experience (see Spence, 2017; Spence et al., 2017), given that most of what we think we taste actually results from retronasal olfaction (see Spence, 2015b).

A few years ago I myself was involved in a mixed reality VR simulation project together with Havana Club rum. Small groups of cocktail makers (c. 14) were invited to take part in the experience/cocktail masterclass. They were each given a branded box (Google glasses) in which to mount their mobile devices. After downloading the relevant content, the assembled mixologists were invited to take a trip through the streets and bars of old Havana. This included digitally presented street scenes, bar scenes, and shots from the Havana Club distillery. This multisensory digital storytelling was combined with the manual delivery of scent (cf. Pells, 2015). So, perhaps best classed as an example of the use of VR in training/storytelling. Synchronized matching audio of people speaking, and Cuban music could also be heard. Rather than using a digital activation, the scent of the fruit stall was delivered by those hosting the event spritzing the air over the mixologists with a fruity scent. Note, once again, that this is difficult to scale. Parts of the content were then introduced into a branded VR bar experience for customers (cf. Benjamin, 2016).

However, in this case, as in the majority of other cases that your author has observed, when a group of users are required to download or access content online at the same time in a public venue issues of insufficient bandwidth nearly always raise their ugly head. Furthermore, with such a mixed reality presentation further challenges arise when it comes to trying to synchronize the scent with the action seen on screen, with minor delays inevitable between the different viewers (cf. Ademoye & Ghinea, 2009).

Another multisensory experiential cocktail experience was offered at the Berkeley Hotel, London (Ellis, 2017). Now, long-since closed, the multisensory cocktail experience, called “Out of the Blue” was delivered by Sensiks (https://www.sensiks.com/; though see also sensoryco4d, 2021, for an alternative), one of the most advanced companies working in the space of delivering multisensory digital experiences. The technology was used to control the delivery of ambient scents, wind, thermal temperature cues, and so on. This was combined with a series of cocktails (all for a cool £200 for four people for one hour). However, while the Sensiks technology has found a market in therapeutic and relaxation settings, it tends to be well-beyond the price point of all but the richest home users (coming in at c. £25,000 for a multisensory booth, the last time I asked). The lack of content/scripts/games for this technology is also likely to limit its appeal. Hence it would seem unlikely that this scent-enabled version of multisensory experience delivery will become a success in the home entertainment market any time soon.

## Conclusions

As this review of the literature has hopefully made clear, there has long been an interest in adding a scented element to various entertainment activities (see also Olofsson et al., 2017), including art galleries/museums (Spence, 2020a), cinemas (Spence, 2020b), and a variety of live performance settings (Spence, 2021a). Furthermore, as we have seen in this review, providing a scented accompaniment to the rides at theme parks is by now a well-established and successful practice both in North America and the UK (e.g., Aggleton & Waskett, 1999; Lukas, 2008; Mack, 2014). It is striking how in the case of theme parks, the scent is seemingly only ever used to provide a semantically congruent backdrop to the experience (often using patented scent delivery technology, such as the smellitizer at Disney venues; see McCarthy, 1986). It is, however, important to recognize that these rides constitute long-term fixtures at the venues, with visitors being moved through the space on the ride; both factors presumably helping to make scent’s incorporation more practical, more cost effective, and ultimately more successful too. That said, I am not aware of any peer-reviewed empirical research that has compared people’s enjoyment on theme park rides as a function of whether or not a scented element has been introduced (cf. Wei et al., 2019).

When it comes to scent in the context of (home-)entertainment, though, the story (or progress) to date has been much more mixed. The introduction of a scented element into (video-/VR-)gaming, although long-promised/anticipated (see Anonymous, 2008; Brooks, 2000), feels as though it has ultimately failed to materialize, at least outside of the confines of the HCI conferences and gaming trade shows (see also Davis et al., 2007). This despite the fact that a number of possible uses for scent in the context of gaming have been suggested, such as the use of semantically congruent smells, the smell of burning rubber in a racing game, or perhaps distracting one’s opponents in multiplayer games by delivering a foul smell to their consoles (Brooks, 2000). “Back in 2013, the microwave popcorn maker Pop Secret experimented with a limited edition kernel-shaped plastic dongle called The Pop Dongle, which when plugged into the audio jack of an iPhone, emitted the smell of popcorn during game play” (Baig, 2016).

While numerous different technological solutions to the digital control of scent delivery have now been presented at the various HCI conferences (such as CHI and SIGGRAPH) they have not reached the mass market (the closest to success perhaps the Scentee plug-in for mobile devices; see Braun et al., 2016; https://scentee.com), though the recently developed FEELREAL (2021) scent-enhanced add-on looks promising in this regard. Here, it is important to dissociate the technical challenges associated with the efficient digital control of scent delivery from the more psychological question of why one should want to introduce scent to such forms of entertainment in the first place (cf. Davis et al., 2007, for a similar stance concerning the uncertain role of olfaction in a gaming context). As the authors conclude:

A digital olfactory game is described and evaluated. The paper may seem to undermine the whole idea of using the olfactory channel, and leaves it an open question how useful olfaction may eventually prove. It is admitted that significant problems await the design of olfactory experiences. (Davis et al., 2007, p. 25)

Although a wide range of digital scent-release technologies exist, most are very limited in terms of the number of olfactory stimuli that they can release. While not an insurmountable problem, it is important to recognize that this does significantly limit the kinds of uses that scent can potentially be put to. It is important to remember that the inability to create specific scents simply by mixing a limited number of odour primitives (in the way that is achieved in a colour printer, say) means that the number of scent cartridges required for any multi-smell offering is likely to be impractical (see Spence et al., 2017). Furthermore, there is also the “fundamental misattribution error” to contend with (again see Spence et al., 2017). As visually dominant creatures (Hutmacher, 2019), it remains questionable whether scent-enabled gaming/storytelling will ever be anything more than a gimmicky niche option to be demo-ed at the games fairs (e.g., Natividad, 2016; cf. Bass, 2015). There is also the phenomenon of “inattentional anosmia” to contend with (Forster & Spence, 2018). This is where people become blind to scents that they would otherwise normally perceive when engaged in a demanding visual task, such as is typically the case for those playing video games.^19^

The idea that the sensory bandwidth of entertainment could be increased is seemingly always enthusiastically covered/endorsed by the tech press (Porges, 2016; Staub, 2011). At the same time, however, no one appears to be asking the more fundamental question of why adding scent should be deemed a good idea in the first place (cf. Ghinea & Ademoye, 2012; Maggioni et al., 2020; Murray et al., 2017); nor does anyone seemingly ask the question of whether the end user will ever be convinced to buy a smell-delivery device in the first place, nor be bothered to source the refills when the scents run out, as they inevitably will (Spence et al., 2017). In your author’s experience, scent’s role in storytelling becomes much more relevant/interesting just as soon as the normally dominant visual sense is removed (see Delaunay, 2014). It is, though, an open question as to whether a videogame without the “video” element would ever be successful commercially. One further unique challenge with gaming, though, is that course of action is unpredictable. Just imagine how soon the novelty of smelling Laura Croft’s sour fearful sweat in the fetid dank air of the dungeon would wear off, should one happen to get stuck on that particular scent-enabled level.

Taking a leaf out of the long history of digitally controlled scent-enabled cinema (see Spence, 2020b, for a review), it could be argued that scent may have more chance of catching on in these other entertainment formats if it goes beyond merely semantically complementing whatever is happening visually. That is, instead of trying to increase immersion by being congruent with the action shown on screen it should be used to tell part of the story, to play jokes on the gamer, and/or perhaps to manipulate their mood (see Spence, 2020b, for a review). There might also be a functional use to scent, especially amongst the most competitive video gamers out there (Bode, 2019; Silady, 2020). Might the release of synthetic chemosensory fear signals, for example, make certain games more exciting, or at the very least improve a player’s performance (Chen et al., 2006)? Releasing the scent of peppermint has also been documented to improve people’s performance in a range of tasks (e.g., Barker et al., 2003; Ho & Spence, 2005; Warm et al., 1991; see also Amores & Maes, 2017). In this case, though, scent might not necessarily need to present a semantically congruent signal but rather just something that delivers a functional benefit to the gamer’s performance. Here it is worth bearing in mind how the video game business was estimated to be worth more than $100 billion back in 2010 (Goldman, 2010).^20^

The limited appeal/success of adding an olfactory element to gaming/home-entertainment (at least thus far) can perhaps be compared to the similar failure of tactile devices, such as the “Butt Shaker” to catch on (once again, except seemingly at the theme park; see Gallace & Spence, 2014; Parisi, 2018; Paterson, 2007; though see also Blenkinsopp, 2019; Porges, 2016). Ultimately, in the absence of a clear user need/benefit/desire on the part of the consumer, it would seem more likely that those introducing scent will do so only when the return on investment is assured (e.g., see Hirsch, 1995; Spence, 2015a) or when there is a demonstrable functional benefit to the user. As such, there are a number of reasons why one might want to disagree with Morton L. Heilig when, in his 1962 article, he provocatively asked: “If we’re going to step through the window into another world, why not go the whole way?”

## Notes

1 Given that listening to music constitutes another especially popular form of home-entertainment (e.g., Bull, 2000; Bull & Black, 2005; North & Hargreaves, 2008), it is perhaps surprising that olfactorily enhanced musical experiences have yet to become popular outside of the nascent space of multisensory experiential/performance (see Di Stefano et al., 2021).

2 Note that this review will not cover scent’s use in the setting of the museum/art gallery (see Spence, 2020a, for a review), nor its use in the cinema (see Spence, 2020b, for a review), nor live-performance settings either (see Spence, 2021a, for a review), given that these topics have recently been reviewed elsewhere.

3 While deLahunta (2003, p. 85) describes the Sensorama stimulator as “a multisensory arcade machine,” which is indeed very much what it looks like (see Figure 1), I would argue that is as much an early example of multisensory storytelling as anything else.

4 This is intriguing given that in the cinematic/theatrical context the release of a pine scent often reminds audiences of a household cleaning product rather than the tree (see Spence, 2020b, 2020c). It will therefore remain for future research to determine whether this reflects a genuine cross-cultural difference in source-object attribution (who knows, perhaps pine-scented cleaning products are simply much less common in Korea than in North America), or whether instead the different response/association elicited speaks to the particular perceptual qualities of the pine scents used in the various situations.

5 Here, though, it is worth noting that lemon is one of those scents that people often associate with cleaning products rather than with the citrus fruit (Holland et al., 2005; see Spence, 2020c).

6 According to Brumfield (2018): “When Disneyland opened in 1955, Walt added a vanilla scent outside the Candy Palace when they weren’t making candy. ‘The faint smell was intended to enhance the feeling that you were on the main street of a real, small town in America’, notes Imagineer Eric Jacobson.”

7 Although one might perhaps want to question the value to those on the rides, of having the scent delivered “fresh.”

8 Apparently, a Chicago firm called Inscentivation Inc. bought the exclusive rights to market the scent to casinos worldwide (Classen et al., 1994).

9 It is easy to imagine how such air fresheners would presumably be especially popular during big competitions such as the European or World Cup.

10 Interestingly, and presciently one might say, the article continues: “Well, bad news, Em. Despite the old Bugs Bunny cartoon (in which a futuristic headline proclaims SMELL-O-VISION REPLACES TELEVISION!), scented TV is still unlikely to be in our parlors 20 years from now. (Emeril, alas, very likely will.) The reason is less technical than economic” (Poniewozik, 2000).

11 According to Milgram and Kishino (1994), mixed reality (MR) is the merging of real and virtual worlds in order to generate new multisensory environments and visualizations, where real and digital objects/stimuli co-exist and can interact in real time. Note that mixed reality does not exclusively take place in either the physical world or the virtual world, but is rather a hybrid reality involving both physical and virtual elements.

12 COLOURED, STEREOSCOPIC FEELY. WITH SYNCHRONIZED SCENTORGAN ACCOMPANIMENT. “Take hold of those metal knobs on the arms of your chair,” whispered Lenina. “Otherwise you won't get any of the feely effects” (Huxley, 1932, p. 119).

13 Here, I am reminded of the following quote: “‘Visionaries,’ says TV analyst Josh Bernoff of Forrester Research, ‘think about what is possible without thinking about what will actually make good business’” (Poniewozik, 2000).

14 Of course, one could say much the same about the much vaunted arrival of purely visual VR, or VR gaming (Adams, 2016).

15 One Reddit thread from 2008 asks why there are no board games that smell (https://www.reddit.com/r/boardgames/comments/28dg2d/board_games_that_use_our_sense_of_smell/). However, a quick online search reveals that there is one game that does smell, called “WowWee What's That Smell? The Party Game That Stinks – Scent Guessing Game For Adults & Families,” but where the identification of the scents is the core component of the game, not simply an augmentation that would function just as well without the scented element.

16 For reference, researchers have demonstrated that adding background Australian rock music (heavy metal) significantly impaired the performance of men, but not women, on the game when compared to listening to the pre-recorded sounds of an operating theatre. The would-be male surgeons in this study were significantly slower while listening to the rock music and there was also a tendency for them to make more surgical mistakes while playing this game (Fancourt et al., 2016).

17 Remember the battle between VHS and Betamax in the playing of videocassette (see Logie, 2020).

18 Luca Turin is a controversial figure in the field of olfaction and fragrance. A biophysicist by training, his name is associated with the vibrational theory of olfaction (see Editorial, 2004).

19 According to a long history of research, olfactory detection thresholds do not tend to be any lower in the congenitally blind than in the sighted, though their ability to discriminate and identify the source of odours is often found to be enhanced (Rosenbluth et al., 2000).

20 Pretty impressive growth when one considers the fact that the very first video game system only entered the market in 1972 (Al-Bahadly & Reda, 2013).
